# Structural and Functional Brain Abnormalities in Internet Gaming Disorder and Attention-Deficit/Hyperactivity Disorder: A Comparative Meta-Analysis

**DOI:** 10.3389/fpsyt.2021.679437

**Published:** 2021-07-01

**Authors:** Xinyu Gao, Mengzhe Zhang, Zhengui Yang, Mengmeng Wen, Huiyu Huang, Ruiping Zheng, Weijian Wang, Yarui Wei, Jingliang Cheng, Shaoqiang Han, Yong Zhang

**Affiliations:** ^1^Department of Magnetic Resonance Imaging, The First Affiliated Hospital of Zhengzhou University, Zhengzhou, China; ^2^Key Laboratory for Functional Magnetic Resonance Imaging and Molecular Imaging of Henan Province, Zhengzhou, China; ^3^Engineering Technology Research Center for Detection and Application of Brain Function of Henan Province, Zhengzhou, China

**Keywords:** internet gaming disorder, attention-deficit/hyperactivity disorder, rewards circuit, voxel-based morphometry, functional connectivity

## Abstract

**Background:** Patients with Internet gaming disorder (IGD) and attention-deficit/hyperactivity disorder (ADHD) have high comorbidity but it is still unknown whether these disorders have shared and distinctive neuroimage alterations.

**Objective:** The aim of this meta-analysis was to identify shared and disorder-specific structural, functional, and multimodal abnormalities between IGD and ADHD.

**Methods:** A systematic literature search was conducted for whole-brain voxel-based morphometry (VBM) and functional magnetic resonance imaging (fMRI) studies comparing people with IGD or ADHD with healthy controls. Regional gray matter volume (GMV) and fMRI differences were compared over the patient groups and then a quantitative comparison was performed to find abnormalities (relative to controls) between IGD and ADHD using seed-based d mapping meta-analytic methods.

**Result:** The meta-analysis contained 14 IGD VBM studies (contrasts covering 333 IGDs and 335 HCs), 26 ADHD VBM studies (1,051 patients with ADHD and 887 controls), 30 IGD fMRI studies (603 patients with IGD and 564 controls), and 29 ADHD fMRI studies (878 patients with ADHD and 803 controls). Structurally, VBM analysis showed disorder-specific GMV abnormality in the putamen among IGD subjects and orbitofrontal cortex in ADHD and shared GMV in the prefrontal cortex. Functionally, fMRI analysis discovered that IGD-differentiating increased activation in the precuneus and shared abnormal activation in anterior cingulate cortex, insular, and striatum.

**Conclusion:** IGD and ADHD have shared and special structural and functional alterations. IGD has disorder-differentiating structural alterations in the putamen and ADHD has alterations in the orbitofrontal cortex. Disorder-differentiating fMRI activations were predominantly observed in the precuneus among IGD subjects and shared impairing function connection was in the rewards circuit (including ACC, OFC, and striatum).

## Introduction

Internet gaming disorder (IGD) is characterized by difficulties in controlling online gaming behaviors, including symptoms such as craving (1, 2), loss of control, and excessive impulsivity (3, 4). Previous studies have indicated that the prevalence estimates of IGD range from 0.3 to 10.8%, depending on the country and age of the population (5–8). Attention-deficiency/hyperactivity disorder (ADHD) has a prevalence of 5–7% (9) and is typically characterized by symptoms of inattention, hyperactivity, and impulsivity (10).

Several comprehensive reviews reported a strong correlation between IGD and ADHD (11). These two disorders share some key features such as impulsivity, seeking immediate rewards, motivation deficit, and hostility (12, 13). A single prospective study followed over 2,000 adolescents for 2 years and found that ADHD was the most significant predictor for the development of internet addiction (14). Moreover, both IGD and ADHD have deficits in the reward circuit, which includes the prefrontal cortex (PFC), anterior cingulate cortex (ACC), orbitofrontal cortex (OFC), striatum (containing the caudate nucleus, putamen, globus pallidus), amygdaloid nucleus, and thalamus (15–17).

Current evidence shows that most addictive diseases exert initial reinforcing effects by activating reward circuits in the brain (18). Weinstein (19) has shown that individuals who are addicted to video-game playing obtain much pleasure during play because of extensive dopamine release. In addition, functional magnetic resonance imaging (fMRI) studies of the reward circuit showed hyperactivity in the bilateral dorsolateral prefrontal cortex (DLPFC), caudate nucleus, the supplementary motor cortex (SMA), and ACC among IGD people (2, 20). Moreover, people with IGD have abnormal structural alterations that include reduced gray matter volume (GMV) in the bilateral ACC, OFC, SMA, right putamen, and left dorsolateral prefrontal cortex through different studies (17, 21, 22).

In addition, Blum et al. (23) showed that ADHD is a reward deficiency disorder, and some theories considered that reward deficiency might predispose individuals to addictive, impulsive, and compulsive behavior. An ADHD, fMRI meta-analyses displayed hypoactivation in the right and left ventrolateral prefrontal cortex (VLPFC), anterior insular (AI), caudate nucleus, middle frontal gyrus (MFG) (24), SMA, and ACC. Moreover, whole-brain voxel-based morphometry (VBM) studies found common decreased GMV in the right globus pallidus and putamen, caudate nucleus, ventromedial prefrontal cortex (VMPFC), and ACC (25–27).

The above studies showed brain structural abnormalities were observed in the cingulate, striatum, frontal, and temporal lobes between these two disorders (15, 17). Moreover, both IGD and ADHD have abnormal whole-brain functional connectivity, such as deficits in the reward circuit (17, 28), although they may show much heterogeneous performance. However, only one study on VBM and no task fMRI compared these two disorders directly. The VBM study (29) showed that IGD subjects with a history of childhood ADHD symptoms had greater GMV in the angular gyrus, middle occipital gyrus, and lingual gyrus than IGD subjects who did not have childhood ADHD symptoms. However, the relatively small sample size of this study is statistically limited. This study aimed to establish the most consistent disorder-differentiating, shared structural, and functional deficits, which are important for developing disorder-specific or transdiagnostic treatment. A comprehensive meta-analysis was conducted, comparing structural and functional abnormalities between IGD and ADHD. Furthermore, multimodal structural and functional abnormalities were performed through conducting conjunction/disjunction analyses across VBM and fMRI studies.

According to previous studies, we hypothesized that disorder-specific GMV abnormality would be shown in the OFC among ADHD subjects (27) and in the putamen in IGD people, whereas we expected disorder-shared decreased GMV in the prefrontal cortex and striatum for both (29). As for fMRI, we hypothesized that IGD-differentiating increased activation in the prefrontal regions (e.g., OFC) (17) where ADHD patients show hypoactivation, and shared abnormal overactivation in the cingulate cortex in both disorders.

## Methods

### Publication Search and Study Inclusion

Systematic and comprehensive searches were performed in the PubMed, Web of Knowledge, and Science Direct databases from January 1, 2010, to October 31, 2020, using different combinations of the keywords “voxel-based morphometry” or “VBM” or “morphometry” or “gray matter” or “functional magnetic resonance imaging” or “fMRI” and “online-game” or “Internet gaming disorder” or “IGD” or “Attention-Deficit/Hyperactivity Disorder” or “ADHD.” We identified further papers by reference tracking and consulting retrieved high-quality meta-analysis and review articles.

The included studies had to meet the following criteria: (1) they provided whole-brain pairwise voxel-based comparisons of patient groups (IGD or ADHD) relative to controls; (2) they were a task-related fMRI or VBM study; (3) they provided peak coordinates in Montreal Neurological Institute (MNI) or Talairach spaces; (4) the diagnosis of ADHD patients had to be based on DSM-IV-TR, or DSM-5, or ICD-10 criteria, and IGD was diagnosed according to DSM-5 or YIAS or CIAS; and (5) there were no neurological or psychiatric comorbidities (such as depression, anxiety, autism, learning disorder, and epilepsy).

We excluded studies that had fewer than 10 patients, those that used only ROI analyses, duplicated patient data, or no eligible contrasts (25, 30–32). If studies did not report peak coordinates, corresponding authors were contacted for necessary details; otherwise, these studies were excluded from the meta-analysis.

The two authors (Gao and Zhang) assessed all articles and achieved 100% agreement.

### Statistical Analysis

We used an anisotropic effect-size version of the Seed-based d Mapping software package (AES-SDM) (version 5.15) to conduct the voxel-wise meta-analysis (https://www.sdmproject.com/), following MOOSE guidelines for meta-analyses of observational studies. The AES-SDM data processing procedure is briefly summarized here (http://www.sdmproject.com/software/tutorial.pdf). AES-SDM uses an anisotropic non-normalized Gaussian kernel to recreate an effect-size map and an effect-size variance map for the contrast between patients and controls from peak coordinates and effect sizes for each VBM or fMRI study. Coordinates were converted to Montreal Neurological Institute (MNI) space for this analysis. Following this, a mean map is created by performing a voxel-wise calculation of the random-effects mean of the study maps, weighted by sample size and variance of each study and between-study heterogeneity. In addition, full width at half maximum (FWHM) was set to 20 mm because this setting was optimal to balance sensitivity and specificity and other parameters included voxel *P* = 0.005, peak height Z = 1, and cluster extent = 10 voxels (33).

First, separate analyses were conducted to examine regional GMV within each patient group (IGD and ADHD) relative to controls and then between the two disorders. Second, fMRI meta-analyses were conducted to examine the neural activation abnormalities observed within and between disorders using all available data. Then a conjunction analysis across both patient groups relative to controls was further performed to examine areas of shared/contrasting abnormalities; This conjunction method was also used within patient groups to conduct multimodal analyses, which showed regions of overlapping functional and structural abnormalities compared with controls. Some studies used multiple task contrasts, several functional tasks, or identical controls. Combined maps with reduced variance were calculated to avoid dependent data in the analyses (26). To examine the effects of age and gender, meta-regression analyses were performed. Finally, we also conducted additional reliability analyses to assess the robustness of the findings: a jackknife sensitivity analysis, which repeated the same analysis excluding one study each time, to assess the reproducibility of the results for each meta-analysis. Moreover, an Egger's test was used to examine possible publication bias.

A statistical threshold *p* < 0.005 was used for all meta-analyses (32, 34), and a reduced threshold *p* < 0.0005 and a cluster extent 20 voxels was used in the meta-regression to control for false positives (35).

## Result

### Search Results and Sample Characteristics

**Figure 1:**
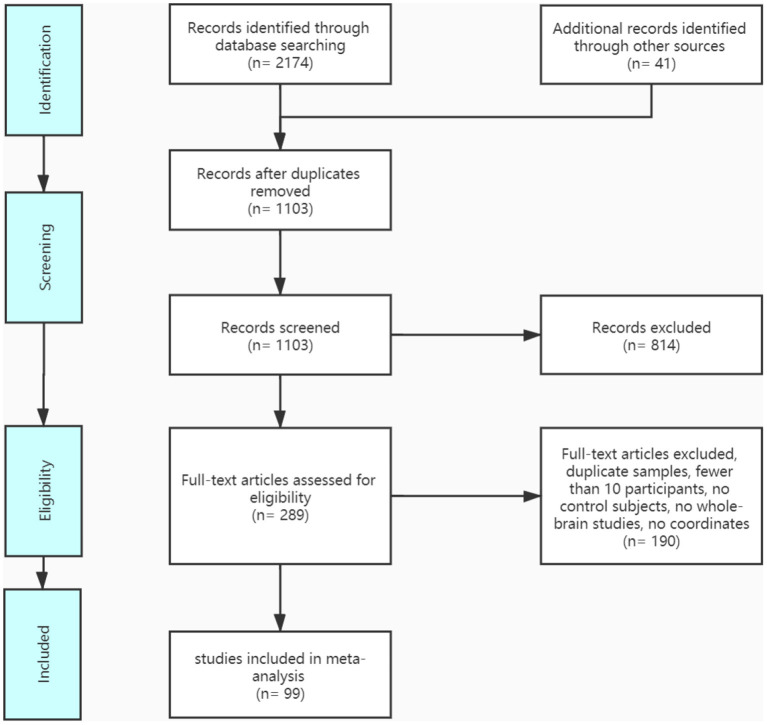
Literature search methods and results for ADHD and IGD fMRI and VBM.

A pool of 2,174 retrieved publications was searched and 41 additional records were identified through other sources. After duplicates were removed, 1,103 records were screened and 289 full-text articles were assessed for eligibility. The final dataset comprised 14 IGD VBM studies (contrasts covering 333 IGDs and 335 HCs), 26 ADHD VBM studies (1,051 patients with ADHD and 887 controls), 30 IGD fMRI studies (603 patients with IGD and 564 controls), and 29 ADHD fMRI studies (878 patients with ADHD and 803 controls). See Figure 1 and Tables 1, 2 for more details.

**Table 1:** Sample characteristics of VBM and fMRI studies in IGD and ADHD group.

**References**	**Age group**	**Patients**		**Controls**	
		**Number (% male)**	**Mean age, y**	**Number (% male)**	**Mean age, y**
**1. VBM studies in IGD**					
Du et al. (36)	Adolescents	25 (100)	17.28	27 (100)	17.48
Han et al. (37)	Adults	20 (100)	20.90	18 (100)	20.90
He et al. (21)	Adults	26 (77) 20	20.69	26 (77)	20.46
Jin et al. (38)	Adults	25 (64) 16	19.12	21 (67)	18.76
Ko et al. (39)	Adults	20 (100)	21.70	20 (100)	22.40
Lee et al. (22)	Adults	30 (100)	23.57	30 (100)	24.23
Lee et al. (29)	Adults	31 (100)	24.00	30 (100)	23.00
Lin et al. (40)	Adults	20 (100)	23.90	20 (100)	22.70
Mohammadi et al. (41)	Adults	35 (100)	22.20	36 (100)	22.28
Seok and Sohn (42)	Adults	29 (100)	23.60	29 (100)	22.70
Sun et al. (43)	Adults	18 (83) 15	20.50	21 (86)	21.95
Weng et al. (44)	Adolescents	17 (24) 4	16.25	17 (12)	15.54
Yoon et al. (45)	Adults	19 (100)	22.90	25 (100)	25.40
Zhou et al. (46)	Adolescents	18 (89) 16	17.23	15 (87)	17.81
**2. VBM studies in ADHD**					
Ahrendts et al. (47)	Adults	31 (65)	31.20	31 (65)	31.50
Amico et al. (48)	Adults	20 (75)	33.60	20 (75)	34.70
Bonath et al. (49)	Adolescents	18 (x)	13.60	18 (x)	14.10
Bralten et al. (50)	Adolescents	307 (68)	17.06	196 (49)	16.66
Gehricke et al. (51)	Adults	32 (81)	25.31	40 (83)	23.93
He et al. (52)	Children	37 (100)	9.90	35 (100)	10.70
Jagger et al. (53)	Children	41 (x)	9.61	32 (x)	9.66
Kappel et al. (54)	Adults	16 (94)	23.50	20 (100)	23.70
	Children	14 (71)	9.80	10 (80)	11.00
Sutcubasi et al. (55)	Adolescents	19 (74)	10.32	18 (67)	10.17
Klein et al. (56)	Adults	25 (36)	66.90	34 (18)	68.90
Kobel et al. (57)	Adolescents	14 (x)	10.43	12 (x)	10.92
Kumar et al. (58)	Children	18 (100)	9.60	18 (100)	9.70
Li et al. (59)	Adolescents	30 (100)	10.30	30 (100)	10.30
Lim et al. (60)	Adolescents	29 (100)	13.80	29 (x)	14.40
Almeida Montes et al. (61)	Adults	20 (50)	28.95	20 (50)	27.57
Moreno-Alcázar et al. (62)	Adults	44 (66)	31.61	44 (66)	32.57
Ramesh and Rai (63)	Adolescents	15 (27)	16.80	15 (27)	16.72
Roman-Urrestarazu et al. (64)	Adults	49 (65)	22.23	34 (57)	22.95
Seidman et al. (65)	Adults	74 (x)	37.30	54 (x)	34.30
Sethi et al. (66)	Adults	30 (63)	33.70	30 (63)	32.60
Shimada et al. (67)	Adolescents	17 (88)	10.29	15 (73)	12.80
van Wingen et al. (68)	Adults	14 (100)	32.00	15 (100)	37.00
Vilgis et al. (69)	Adolescents	33 (100)	12.58	31 (100)	12.75
Villemonteix et al. (70)	Adolescents	38 (58)	10.40	25 (60)	10.10
Wang et al. (71)	Adolescents	30 (63)	10.60	25 (48)	10.60
Zhao et al. (72)	Adolescents	36 (x)	12.14	36 (x)	11.69
**3. fMRI studies in IGD**					
Chiao et al. (73)	Adults	15 (100)	24.70	15 (100)	24.47
Chun et al. (74)	Adolescents	16 (100)	13.60	19 (100)	13.37
Dieter et al. (75)	Adults	15 (87)	28.7	17 (76)	24.94
Ding et al. (13)	Adolescents	17 (82)	16.40	17 (82)	16.29
Dong et al. (76)	Adults	18 (100)	21.00	19 (100)	21.00
Dong et al. (76)	Adults	27 (x)	21.00	43 (x)	21.47
Dong et al. (77)	Adults	16 (100)	21.40	15 (100)	22.10
Dong et al. (78)	Adults	14 (100)	23.40	13 (100)	24.10
Dong and Potenza (79)	Adults	20 (100)	21.30	16 (100)	21.90
Dong et al. (80)	Adults	15 (100)	21.60	15 (100)	22.40
Han et al. (81)	Adolescents	15 (100)	14.20	15 (100)	14.00
Kim et al. (82)	Adolescents	13 (x)	14.50	10 (x)	14.20
Ko et al. (83)	Adults	15 (100)	24.70	15 (100)	24.47
Ko et al. (84)	Adults	26 (100)	24.60	23 (100)	24.35
Lee et al. (85)	Adults	24 (100)	24.80	24 (100)	24.3
Lee et al. (86)	Adolescents	18 (18)	13.60	18 (100)	13.40
Lemenager et al. (87)	Adults	16 (88)	28.30	17 (76)	24.94
Lin et al. (88)	Adults	19 (100)	22.20	21 (100)	22.80
Liu et al. (2)	Adults	39 (100)	22.60	23 (100)	23.09
Liu et al. (89)	Adults	11 (100)	23.50	11 (100)	22.45
Liu et al. (90)	Adults	41 (100)	21.90	27 (100)	22.74
Lorenz et al. (91)	Adults	8 (100)	25.00	9 (100)	24.80
Ma et al. (92)	Adults	29 (100)	22.60	23 (100)	23.09
Qi et al. (93)	Adolescents	23 (100)	17.30	24 (100)	17.42
Qi et al. (94)	Adolescents	24 (100)	17.20	24 (100)	17.42
Shin et al. (95)	Adults	20 (x)	22.10	21 (x)	22.14
Sun et al. (20)	Adults	10 (100)	20.40	10 (100)	20.30
Wang et al. (96)	Adults	20 (100)	21.00	20 (100)	21.95
Zhang et al. (97)	Adults	19 (100)	22.20	21 (21)	22.80
Zhang et al. (98)	Adults	40 (100)	22.00	19 (100)	22.89
**4. fMRI studies in ADHD**					
Cubillo et al. (99)	Adults	11 (100)	29.00	10 (100)	28.00
Dibbets et al. (100)	Adults	15 (100)	28.90	14 (100)	28.80
Kooistra et al. (101)	Adults	11 (100)	21.50	11 (100)	22.30
Passarotti et al. (102)	Adolescents	11 (55)	13.10	15 (47)	14.13
Cubillo et al. (103)	Adults	11 (100)	29.00	15 (100)	28.00
Rubia et al. (104)	Adolescents	12 (100)	13.00	13 (100)	13.00
Rubia et al. (105)	Adolescents	12 (100)	13.00	13 (100)	13.00
Spinellli et al. (106)	Adolescents	13 (69)	10.60	17 (47)	10.50
Ma et al. (107)	Children	15 (53)	9.82	15 (53)	9.91
Sebastian et al. (108)	Adults	20 (55)	33.30	24 (46)	30.30
Siniatchkin et al. (109)	Children	17 (82)	9.30	14 (71)	9.10
Bhaijiwala et al. (110)	Adolescents	12 (100)	13.80	12 (100)	15.40
Chantiluke et al. (111)	Adolescents	18 (100)	13.40	25 (100)	14.30
Cubillo et al. (112)	Adolescents	19 (100)	13.00	29 (100)	13.00
Schulz et al. (113)	Adults	14 (100)	23.30	14 (100)	22.80
Chen et al. (114)	Adults	29 (100)	24.90	25 (100)	25.64
Janssen et al. (115)	Adolescents	21 (90)	10.60	17 (76)	10.28
Rasmussen et al. (116)	Adults	25 (68)	24.60	12 (50)	24.10
Van Rooij et al. (117)	Adolescents	185 (70)	17.30	124 (44)	16.50
Ma et al. (118)	Adolescents	25 (76)	15.40	33 (67)	15.30
Zamorano et al. (119)	Adolescents	17 (100)	11.60	17 (100)	11.70
Fan et al. (120)	Adolescents	27 (89)	12.10	27 (70)	13.00
Shang et al. (121)	Adults	25 (56)	28.50	30 (50)	28.17
Thormton et al. (122)	Adolescents	20 (90)	12.40	20 (40)	10.55
Materna et al. (123)	Adults	30 (63)	31.40	35 (54)	28.89
Mehren et al. (124)	Adults	20 (100)	31.40	20 (100)	29.50
Yang et al. (125)	Adults	20 (45)	26.90	20 (40)	27.70
Ariadna et al. (126)	Adolescents	18 (67)	10.30	14 (64)	11.12
Pretus et al. (127)	Adults	21 (52)	36.50	24 (50)	34.33

*WHO, Children:1-9y; Adolescents, 10-19y; Adults, >19y*.

**Table 2:** Demographic information for studies included in meta-analysis.

**Characteristic**	**IGD**	**ADHD**	**IGD controls**	**ADHD controls**
**Voxel-based morphometry**				
Patients, no.	333	1,051	335	887
Male sex, no. (%)	300 (90)	811 (77)	302 (90)	639 (72)
Mean age, y	21.3	20.3	21.43	21.3
**Functional magnetic resonance imaging**				
Patients, no.	603	878	564	803
Male sex, no. (%)	596 (99)	540 (62)	553 (98)	448 (56)
Mean age, y	21.3	15.4	21.2	15.8

In the VBM analysis, Wilcoxon W tests revealed that patient groups did not differ in age (*z* = −1.155; *P* = 0.248), and Chi-squared test showed both groups contained a significantly greater proportion of males (χ^2^ = 26.362; *P* = 0.001). In the fMRI meta-analysis, patient groups did not differ in age (*z* = −1.077; *P* = 0.282) but a large proportion of patients with IGD and ADHD were males (χ^2^ = 93.565; *P* = 0.001). Age and sex were consequently included as covariates in all between-group meta-analyses performed including only the adult studies, which were age and sex matched.

### Disorder-Differentiating and Shared Brain Structure Abnormalities

#### Regional Differences in GMV

##### IGD VBM

**Figure 2:**
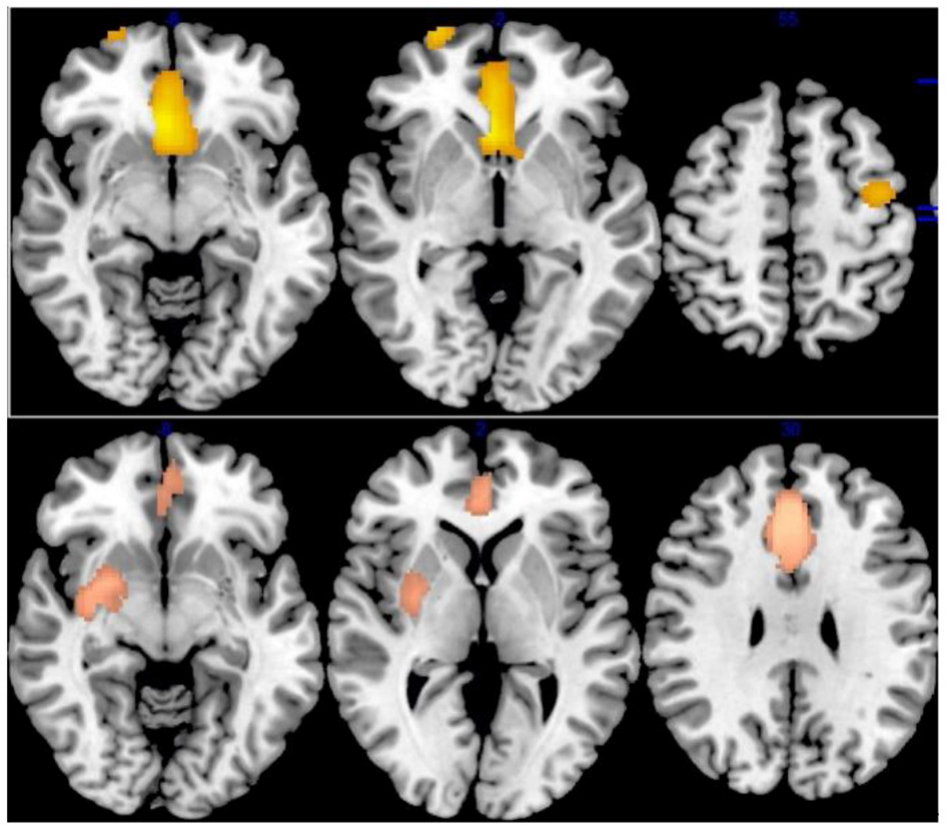
Results of Voxel-Based Morphometry (VBM) for IGD and ADHD. Results of VBM meta-analysis for, from top to bottom, patients with Internet gaming disorder (IGD) relative to controls, patients with attention-deficit/hyperactivity disorder (ADHD) relative to controls.

Relative to healthy controls, IGD had reduced gray-matter volume (GMV) in the bilateral anterior cingulate cortex (ACC), median cingulate cortex (MCC), superior frontal gyrus (SFG), the supplementary motor cortex (SMA), right putamen/striatum, bilateral inferior frontal gyrus (IFG), and left middle frontal gyrus (MFG) (Table 3 and Figure 2).

**Table 3:** Meta-analysis results for voxel-based morphometry studies in IGD and ADHD.

**Contrast**	**MNI coordinates**			**SDM Z score**	***P*-value**	**Voxels number**	**Jack-knife sensitivity**	**Brodmann areas**
	***X***	***Y***	***Z***					
**1. VBM RESULTS**								
**1) IGD decreased vs. control**								
L ACC/R ACC/L SFG/R MCC/L MCC/R SMA/L SMA/R SFG	0	40	12	−2.636	0.000005186	2,107	14 out of 14	32, 24, 10, 11, 6
R putamen/R striatum	28	0	−4	−2.277	0.000072241	392	14 out of 14	48
L MFG/L IFG	−42	34	18	−1.742	0.001615345	99	11 out of 14	45, 46
R IFG	50	32	18	**-**1.599	0.003282249	20	11 out of 14	45
**2) ADHD decreased vs. control**								
L ACC/L SFG/R SFG/R ACC/L olfactory cortex/R olfactory cortex/L median cingulate/L caudate/L striatum/R striatum/R MCC/L median network/R gyrus rectus/L gyrus rectus	4	24	–	−3.037	0.000361264	1,849	25 out of 27	10, 11, 24, 25, 32
L precentral gyrus/L postcentral gyrus	−40	−6	54	−2.248	0.000743151	176	26 out of 27	6
R OFC/R DLSFG	26	68	−2	−2.381	0.000407696	92	26 out of 27	11
L OFC	−24	16	−24	−2.222	0.000830889	55	25 out of 27	38
**3) IGD (vs. control) vs. ADHD (vs. control)**								
IGD (vs. control) decreased vs. ADHD (vs. control)								
R striatum	28	−4	−10	1.958	0.000103235	24		
ADHD (vs. control) decreased vs. IGD (vs. control)								
L caudate nucleus	−12	8	6	1.626	0.000030994	89		
**2. fMRI RESULTS**								
**1) IGD increased vs. control**								
L precuneus/R precuneus/R MCC/L MCC/R PCC/L PCC	−4	−58	38	2.253	0.000139356	1,185	30 out of 30	7, 23
R IFG/R precentral gyrus	50	12	16	2.454	0.000015497	808	30 out of 30	6, 44, 45, 48
L angular gyrus/L MTG/L MOG	−42	−64	28	2.098	0.000376761	465	30 out of 30	19, 39
L precentral gyrus/L IFG	−48	8	30	2.167	0.000232220	189	28 out of 30	6, 44
R caudate nucleus	10	8	18	1.819	0.001832068	33	24 out of 30	25
IGD decreased vs. control								
R precentral gyrus/R MFG/R postcentral gyrus	42	−10	48	−1.489	0.000092924	831	27 out of 30	3, 4, 6
R insula/R Rolandic operculum	34	−20	18	−1.391	0.000175476	295	28 out of 30	48
L precentral gyrus	−38	−24	68	−1.209	0.000547051	111	28 out of 30	4, 6
**2) ADHD increased vs. control**								
R MFG/R DLSFG	18	48	28	1.166	0.000392199	103	27 out of 29	9, 46
R ACC	12	42	22	1.158	0.000030994	52	28 out of 29	32
ADHD decreased vs. control								
R precentral/R postcentral gyrus	30	−24	54	−3.584	~0	813	29 out of 29	2, 3, 4, 6
L STG/L insula/L OFC	−46	12	20	−3.167	0.000113547	286	28 out of 29	38, 47
R STG/R MTG	56	−40	10	−3.172	0.000113547	147	28 out of 29	22, 42
L DLSFG/L MFG	−24	38	36	−3.246	0.000072241	55	28 out of 29	9, 46
**3) IGD (vs. control) vs. ADHD (vs. control)**								
ADHD (vs. control) decreased vs. IGD (vs. control)								
L MCC	−2	−6	32	−3.534	0.000010312	254		23, 24
R STG	52	−40	12	−2.987	0.000836074	88		41, 42
R caudate nucleus	10	8	20	−3.392	0.000046432	57		
L MFG	−20	46	32	−2.925	0.001109600	20		9
IGD (vs. control) decreased vs. ADHD (vs. control)								
Null								
**4) Multimodal analysis in IGD**								
VBM increased and fMRI increased								
R MOG/angular gyrus	38	−70	36	1		456		7, 19, 39
R precuneus	8	−56	34	1		258		23
VBM decreased and fMRI increased								
L ACC/SFG	4	16	22	1		832		24,32
R IFG	50	24	24	1		445		45,48
VBM decreased and fMRI decreased								
Insula/putamen	36	−8	4	1		240		48
**5) Multimodal analysis in ADHD**								
VBM increased and fMRI increased								
R fusiform gyrus	34	−8	−28	1		39		20
VBM decreased and fMRI increased								
R MCC	4	24	32	1		1,315		24
R SFG(OFC)	4	38	−14	1		766		11
R STG	62	−12	4	1		376		22
R MOG	40	−82	6	1		229		19
VBM decreased and fMRI decreased								
R STG	58	−42	16	1		660		42
L IFG	−30	16	−24	1		543		38
L postcentral gyrus	−50	−14	46	1		222		4

*ACC, anterior cingulate cortex; DL, dorsolateral; IFG, inferior frontal gyrus; L, Left; MNI, Montreal Neurological Institute; MCC, median cingulate cortex; MFG, middle frontal gyrus; MOG, middle occipital gyrus; MTG, middle temporal gyrus; OFC, orbitofrontal cortex; PCC, posterior cingulate cortex; R, Right; SDM, seed-based d mapping; SMA, supplementary motor area; SFG, superior frontal gyrus; STG, superior temporal gyrus*.

##### ADHD VBM

ADHD Patients compared with HCs showed significantly lower GMV in the bilateral ACC/olfactory cortex/median cingulate, bilateral striatum, left caudate nucleus, left precentral gyrus/postcentral gyrus, right superior frontal gyrus, and orbitofrontal cortex (OFC) (Table 3 and Figure 2).

##### IGD vs. ADHD VBM

People with IGD, relative to ADHD, had more reduced GMV in the right striatum (Montreal Neurological Institute [MNI]coordinates, 28, −4, −10; 44 voxels); while people with ADHD showed lower left caudate nucleus GMV (MNI coordinates, −12, 8, 6; 89 voxels), relative to IGD (Table 3).

### Disorder-Differentiating and Shared Brain Functional Connectivity

#### IGD fMRI

**Figure 3:**
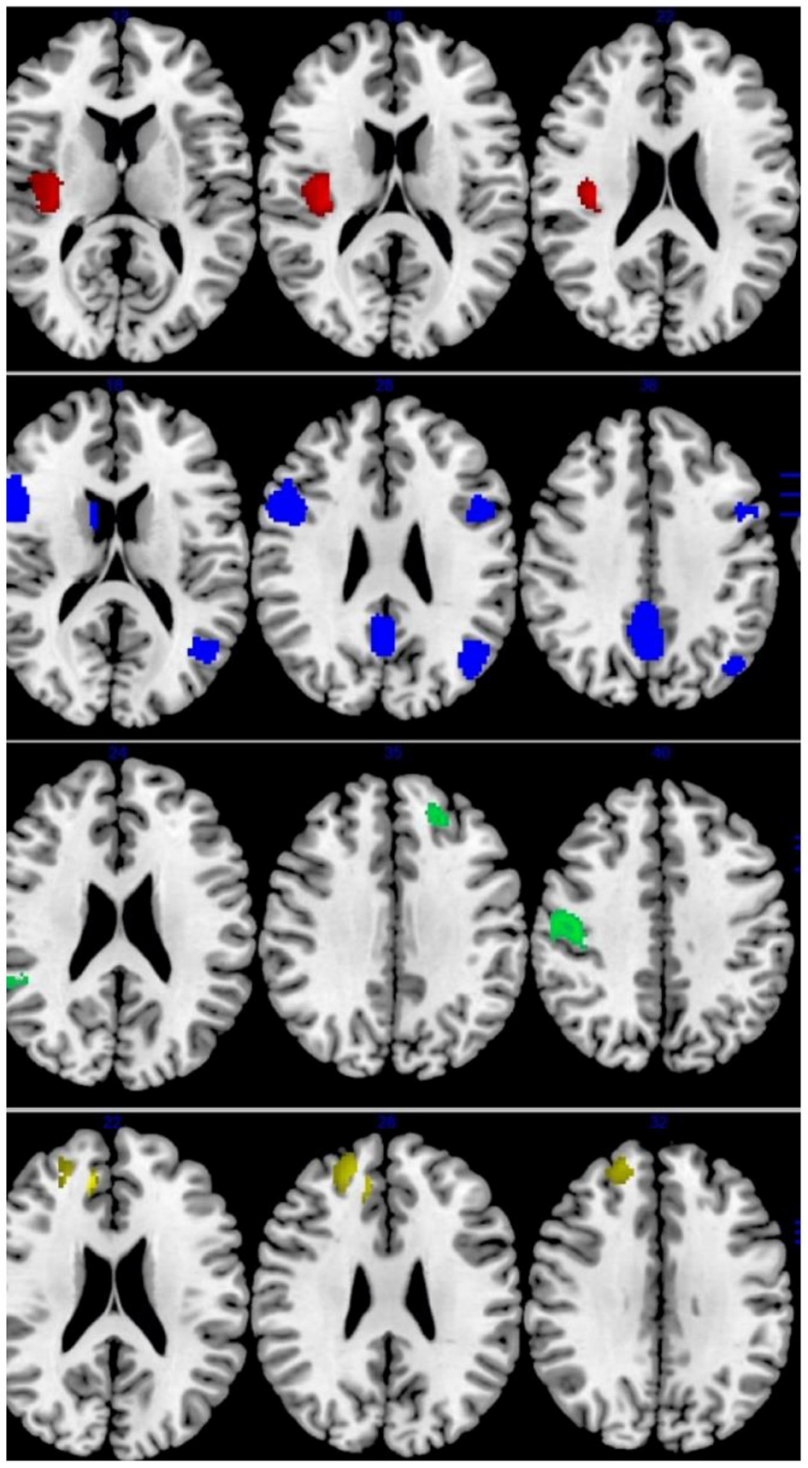
Results of Functional Magnetic Resonance Imaging (fMRI) for IGD and ADHD. Results of fMRI meta-analysis for, from top to bottom, patients with Internet gaming disorder (IGD) relative to controls (red: increased in IGD; blue: decreased in IGD), patients with attention-deficit/hyperactivity disorder (ADHD) relative to controls (green: increased in ADHD; yellow: decreased in ADHD).

Across all fMRI studies, people with IGD showed activation in the bilateral precuneus/cingulate cortex (CC), right OFC, left angular gyrus/middle temporal gyrus (MTG)/MOG, left precentral gyrus, bilateral IFG, right caudate nucleus. Moreover, IGDs had lower activation in the right precentral and postcentral gyri, right insular/rolandic operculum, compared with controls (Table 3 and Figure 3).

#### ADHD fMRI

Patients with ADHD relative to controls showed overactivation in the right DLPFC, right MFG, and right ACC. Hypoactivation was observed in the right precentral gyrus (motor cortex), left STG/insula/OFC, right STG/MTG, left DLPFC/MFG (Table 3 and Figure 3).

#### IGD vs. ADHD fMRI

ADHD was associated with disorder-specific hypoactivation relative to IGD in the L MCC, R MTG, R caudate nucleus, and L MFG (Table 3).

### Multimodal VBM and fMRI Analyses

#### Multimodal Analysis in IGD

**Figure 4:**
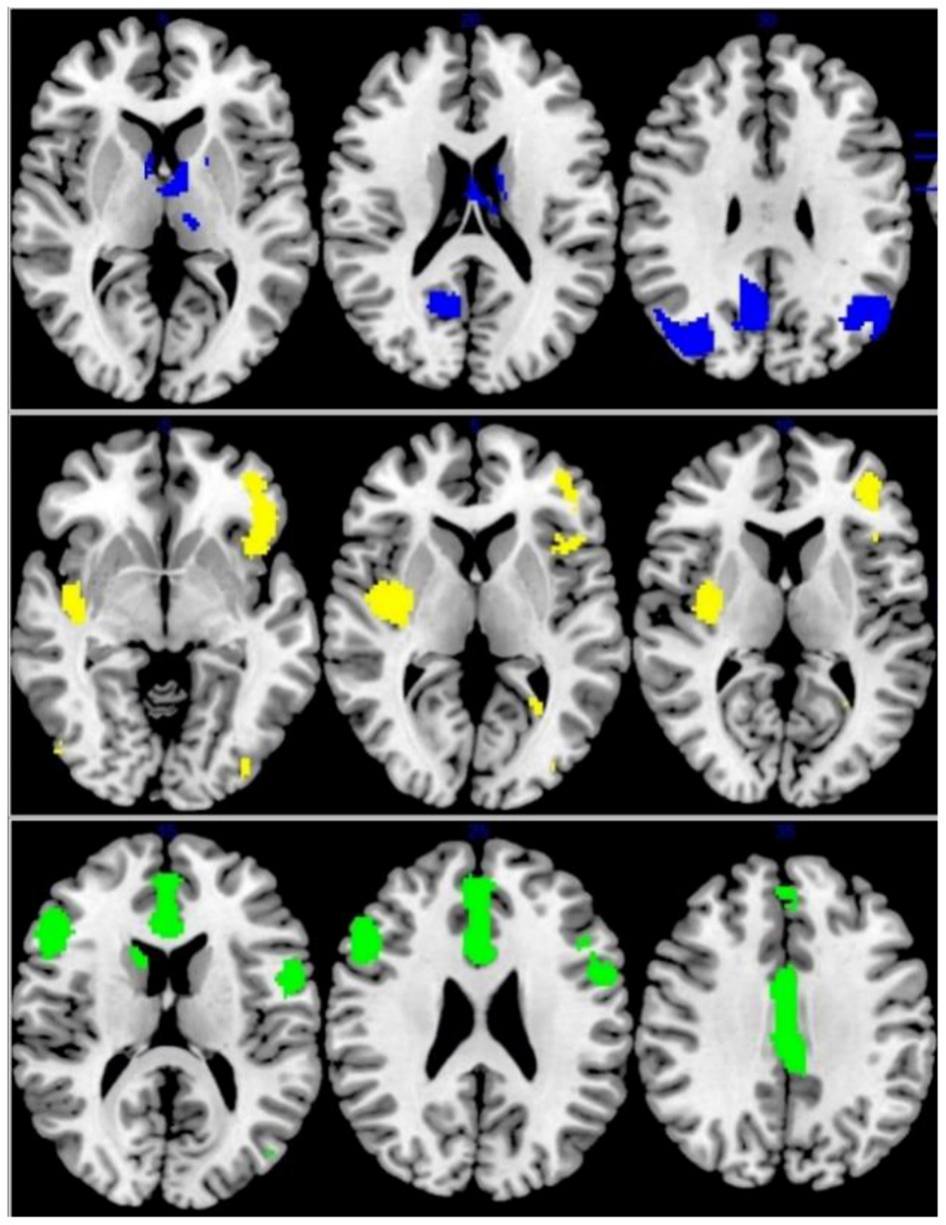
Multimodel analysis in the IGD. Blue: increased GMV and increased activation; Yellow: decreased GMV and functional connection; Green: decreased GMV and increased activation.

In patients with IGD, decreased GMV and functional connection relative to controls overlapped in the right insular/putamen (MNI coordinates, 36, −8, 4; 240 voxels) while increased GMV overlapped with increased activation in the right angular gyrus/MOG and precuneus (MNI coordinates, 38, −70, 36 and 8, −56, 34; 456 voxels and 258 voxels, respectively). The left ACC and right IFG was decreased in volume and increased in function connection in patients with IGD relative to controls (MNI coordinates, 4, 16, 22 and 50, 24, 24; 832 and 445 voxels) (Figure 4).

#### Multimodal Analysis in ADHD

**Figure 5:**
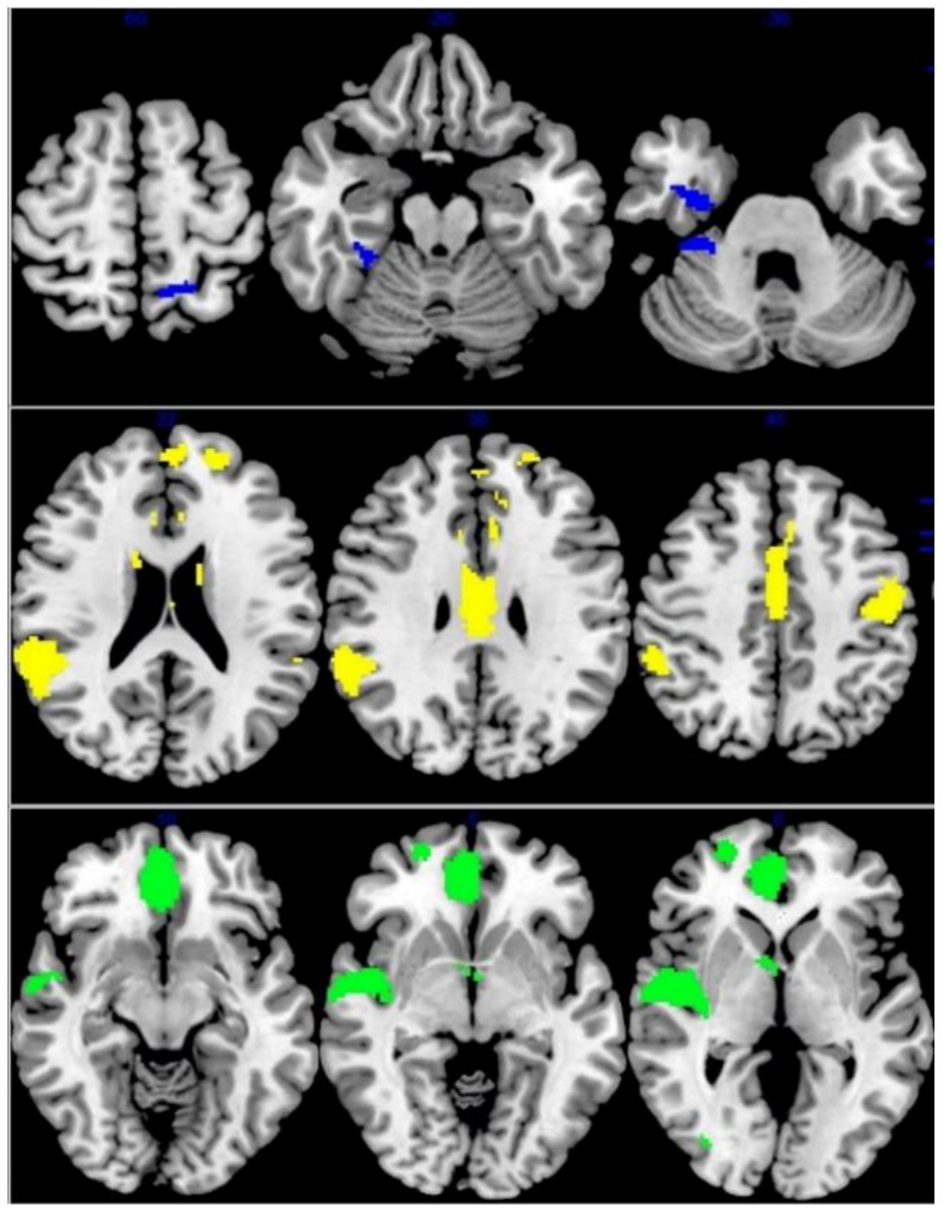
Multimodel analysis in the ADHD. Blue: increased GMV and increased activation; Yellow: decreased GMV and functional connection; Green: decreased GMV and increased activation.

As for ADHD patients, increased GMV and functional activation relative to controls overlapped in the right fusiform gyrus (MNI coordinates, 34, −8, −28; 39 voxels) while decreased GMV overlapped with decreased activation in the right superior temporal gyrus, left inferior frontal gyrus, and left postcentral gyrus (MNI coordinates: 58, −42, 16; −30, 16, −24 and −50, −14, 46; 810 voxels; 776 voxel and 222 voxels, respectively) (Figure 5).

### Publication Bias

Egger's tests were performed to examine potential publication bias. The results of the Egger tests were non-significant (*P* > 0.05 for all comparisons, Bonferroni corrected), suggesting that there was no publication bias. Jack-knife reliability analyses suggested robust disorder-differentiating findings.

## Discussion

The purpose of our meta-analytic comparison is to show that patients with IGD and ADHD have predominantly shared and disorder-specific patterns of structural and functional abnormalities, especially in reward function. Structurally, IGD people have decreased putamen GMV and ADHD patients have lower GMV in the orbitofrontal cortex. Functionally, precuneus was reported as disorder-special activation in IGD patients. Furthermore, functional alteration in the OFC was opposite, which is activated in IGDs and hypoactive in ADHD. Moreover, disorder-specific increased GMV and functional activation were found in the precuneus among IGD patients and in the fusiform gyrus in ADHD patients through multimodal analysis. Patients with IGD and ADHD showed commonly the same direction of change in the ACC (decreased GMV and hyperactivation) and insula (decreased GMV and lower activation). In addition, striatum, expecting abnormal structure in both two disorders, was reported to be reduced in GMV and functional connectivity in the IGD group and reduced in GMV and no significant change in fMRI for the ADHD group.

The key disorder-shared abnormality in two disorders both in structure and function is the prefrontal-striatum circuit. The circuit network contains the anterior cingulate cortex, the orbital prefrontal cortex, the ventral striatum, the ventral pallidum, the dorsal prefrontal cortex, amygdala, hippocampus (16). Attention-deficit/hyperactivity disorder (ADHD) has been conceptualized as a disorder of the prefrontal cortex for over 30 years (28) and IGD is found to be defective in PFC through various studies (22, 40), our results showed that parts of structural and functional alterations in ADHD and IGD patients concentrated on PFC areas. The main cortical areas in the PFC areas associated with reward are the anterior cingulate cortex and orbitofrontal cortex.

As predicted, the results of the main meta-analyses converged on the ACC, which showed functional hyperactivation and gray-matter reduction in IGDs and ADHD relative to HCs. This finding is in line with recent transdiagnostic meta-analyses that this region may serve as a common bio-marker across psychiatric disorders (128), possibly because it modulates the neural activity of the default-mode network and executive control network (129) and is critically involved in multiple processes including cognitive control (130), emotional regulation (131), and reward-relative decision-making (132). Bonath et al. found significantly smaller ACC gray matter volume in subjects with ADHD and reduced volume in ACC was directly associated with symptoms of attentional deficits (49). IGD participants cannot control their compulsion to play Internet games despite experiencing negative consequences due to impaired cognitive control of ACC (40). These studies are consistent with our findings. Neural alterations in the ACC area, consistent with the interaction of the Person-Affect-Cognition-Execution model, play an important role in cue-induced craving, rewards-seeking, (22) and cognitive control in the ACC among IGD and ADHD patients. Moreover, although the direction of the alteration of the ACC is inconsistent across modalities (fMRI and VBM), previous evidence suggests that gray-matter-volume increases or reductions may not simply correspond to functional neural activation or deactivation (132). In conclusion, fMRI and VBM may reflect the distinctive aspects of neural alterations, and the evidence converges to emphasize an important role for the ACC in IGD and ADHD.

The disorder-contrasting findings in OFC are worth discussion. Now it is commonly understood that the OFC contributes to psychotic dysfunction including impulse control and monitoring ongoing behavior and rewards-seeking behaviors (44). Our decreased OFC activation in ADHD is consistent with a previous study that showed decreased cognitive capacity, which is related to hyperactivity and impulsivity and is associated with reduced OFC activity during reward expectation in ADHD patients (133). Furthermore, the strong activation of OFC in IGD patients might be explained by pleasant objects and rewarding anticipation, which refers to internet games in IGD, so that IGD people are more eager to look for stimulation and rewards.

As for the striatum, which comprises the caudate nucleus and the putamen, the nucleus accumbens (NAc), and the olfactory tubercle, which appear in our result. When IGD or ADHD patients are exposed to cue-relative stimulation, the activation of glutamatergic projections from the ventral PFC, the ventral hippocampus, and the amygdala (and presumably medial thalamus) to striatal projections that increase DA signaling and release in the NAc and dorsal striatum will enhance reward craving and eventually result in game activity in IGD and distraction in ADHD (18). However, we found reduced striatum GMV in the IGD and ADHD group and lower functional connectivity in the IGD group, but there was no significant change in fMRI for the ADHD group. Using all kinds of checkout, there was still no significant functional connection alteration in striatum among ADHD patients. We speculate that the reasons could include ADHD fMRI studies that claim there was inconsistent striatum action, meaning there was no result when putting these studies together to conduct meta-analyses.

We found consistent changes in the insula, which had decreased GMV and lower activity in ADHD and IGD subjects. The insula are involved in motivation, rewards, salience detection, and cognitive control (98, 134, 135), modulated by dopaminergic activity (134), which is typically decreased in IGD and ADHD. Therefore, the insula is hypothesized to be a neural system that increases reward drivers and weakens cognitive control (136). In ADHD, deficient insula activation may result in reduced task-related salience detection and cognitive control, resulting in lower self-control ability and increased distractibility. IGD hypoactivation in the insula probably shows that they are habituated to gaming-related stimulation and insensitive to other conventional stimulation, which contributes to gaming addiction.

We find decreased putamen GMV in IGD people through VBM analysis. The dorsolateral putamen has been functionally linked to the sensorimotor cortices, forming the sensorimotor network. A recent research report that health controls show a significant positive correlation in the neural pathways connecting the putamen-MFG-insula when facing gaming cues, which is missing in individuals with IGD (136). Meanwhile, this study also demonstrated increased excitatory neuromodulation in the effective connections among the insula-putamen-OFC in IGD, a neural pathway involving reward-related activity. In conclusion, the putamen is part of the reward pathway, the declination of putamen gray matter may impact its function, which is part of the reason for game addiction.

In our study, disorder-specific activation is suggested in the precuneus among IGD patients. The precuneus is associated with visual imagery, attention, and memory retrieval by participating in the visual process and integrates related memory (137). A possible explanation is that high activation in the precuneus is relative to gaming urge, craving, and the severity of Internet addiction. This result suggests that the precuneus activates to process the gaming cue, and contributes to the cue-induced craving for online gaming. Furthermore, ADHD patients have special activation in the fusiform gyrus. The findings are consistent with a study of reward effect on brain structure and function in adults and children with ADHD (54). The fusiform gyrus (FG), which topographically connects the striate cortex to the inferior temporal lobe, plays a pivotal role in high-level visual/cognitive functions (138). Speculation is that fusiform mediate various stimuli that result in it being hard for people with ADHD to focus on what they are doing.

## Limitation

This meta-analysis has several limitations. First, it was based primarily on peak coordinates rather than raw statistical brain maps. Besides, the heterogeneity of the methodologies among VBM studies could not be avoided, such as the differences in MRI machines, slice thickness, preprocessing protocols (traditional or optimized), and smoothing kernel size, which might have contributed to the inconsistent results (33). Moreover, the included studies have different proportions of males and diverse statistical thresholds, which may lead to discrepant results. Previous studies suggest that neural alterations in some regions may be more severe in female IGDs and ADHDs (17), but future studies are needed to shed more light on gender difference and conduct further research.

## Conclusion

The comparative meta-analytic findings of this study stress the shared and distinctive brain structure and function in IGD and ADHD. Disorder-differentiating structure alterations are reported in the putamen for IGD and in the orbitofrontal cortex for ADHD subjects. Disorder-differentiating fMRI activation was predominantly observed in the precuneus among IGD subjects. The shared functional alterations focus on the frontal-striatum reward circuit, which is important for understanding the underlying pathophysiology and proves that these two disorders have a common neurological foundation. Disorder-shared neurofunctional biomarkers provide useful evidence that the drugs treat ADHD could be used on IGD. Disorder-specific neurofunctional biomarkers could ultimately aid in the development of future, disorder-differentiated behavioral, pharmacological, or neurotherapeutic treatments.

## Data Availability Statement

The original contributions presented in the study are included in the article, further inquiries can be directed to the corresponding authors.

## Author Contributions

XG and YZ designed the experiment. XG, MZ, and ZY performed the experiment. MW, HH, RZ, WW, YW, JC, SH, and YZ modified the experiment and paper. All authors contributed to the article and approved the submitted version.

## Conflict of Interest

The authors declare that the research was conducted in the absence of any commercial or financial relationships that could be construed as a potential conflict of interest.
